# Polycomb represses a gene network controlling puberty via modulation of histone demethylase *Kdm6b* expression

**DOI:** 10.1038/s41598-021-81689-4

**Published:** 2021-01-21

**Authors:** Hollis Wright, Carlos F. Aylwin, Carlos A. Toro, Sergio R. Ojeda, Alejandro Lomniczi

**Affiliations:** Division of Neuroscience, Oregon National Primate Research Center/OHSU, Beaverton, OR USA

**Keywords:** Neuroendocrine diseases, Cellular neuroscience, Epigenetics in the nervous system, Molecular neuroscience

## Abstract

Female puberty is subject to Polycomb Group (PcG)-dependent transcriptional repression. *Kiss1*, a puberty-activating gene, is a key target of this silencing mechanism. Using a gain-of-function approach and a systems biology strategy we now show that EED, an essential PcG component, acts in the arcuate nucleus of the hypothalamus to alter the functional organization of a gene network involved in the stimulatory control of puberty. A central node of this network is *Kdm6b*, which encodes an enzyme that erases the PcG-dependent histone modification H3K27me3. *Kiss1* is a first neighbor in the network; genes encoding glutamatergic receptors and potassium channels are second neighbors. By repressing *Kdm6b* expression, EED increases H3K27me3 abundance at these gene promoters, reducing gene expression throughout a gene network controlling puberty activation. These results indicate that *Kdm6b* repression is a basic mechanism used by PcG to modulate the biological output of puberty-activating gene networks.

## Introduction

A central event in the neuroendocrine cascade leading to the acquisition of reproductive maturity is an increase in pulsatile luteinizing hormone (LH) release from the pituitary gland^1^. This increase is elicited by a change in the episodic discharge of gonadotropin hormone releasing hormone (GnRH), a decapeptide secreted by neurosecretory neurons located in the basal forebrain. In turn, GnRH release is driven by coordinated alterations in trans-synaptic and glial input to GnRH neurons^2,3^. Before puberty, GnRH neuron pulse frequency is kept low predominantly by trans-synaptic inhibitory control provided by GABA^4,5^, opioid^6–10^ and RFamide-related peptide^11,12^ containing neurons. At the end of the juvenile period, increased GnRH pulse frequency is achieved due to diminished inhibitory control as well as increased excitatory trans-synaptic inputs provided by glutamatergic^1,13^ neurons and a family of peptides, known as kisspeptins^14–17^, that robustly stimulate the GnRH network.

All kisspeptins are the product of proteolytic digestion of a 145 amino acid precursor encoded by the *KISS1/Kiss1* gene^18,19^. In female rodents there are two distinct populations of Kisspeptin neurons, one located in the anteroventral periventricular nucleus (AVPV)^20^ and the other in the arcuate nucleus (ARC) of the hypothalamus^21,22^. ARC Kisspeptin neurons are known as KNDy neurons because they produce and release Kisspeptin, Neurokinin B (NKB) and Dynorphin. KNDy neurons release NKB, that acts on other KNDy cells, further stimulating kisspeptin release. Moreover, rhythmic kisspeptin and NKB release is primarily determined by the inhibitory effect of dynorphin on NKB release^21–23^. It is proposed that this cellular feedback loop of activation followed by inhibition in KNDy neurons is the main component of a controlling cellular system termed the GnRH “pulse generator”^24,25^ that plays a central role in the initiation of puberty. While circulating estrogen (E2) inhibits *Kiss1* expression in KNDy neurons, AVPV *Kiss1* expression is strongly activated during the preovulatory surge of gonadotropins, when circulating E2 levels are high^20,26^. It is also known that AVPV kisspeptin neurons innervate ARC KNDy neurons, possibly playing a role in pubertal onset^26^.

Earlier, we described an epigenetic mechanism of transcriptional repression that, operating in the ARC, plays a significant role in timing female puberty^27^. Our results identified the Polycomb group (PcG) of transcriptional silencers^28–30^ as a major contributor to this repressive mechanism. The PcG system is composed of three complexes (PRC1, PRC2 and PhoRC) that work together to silence gene expression^31–33^. The PRC1 complex contains different chromodomain (CBX) proteins^31–33^. The PRC2 complex is composed of four core subunits: enhancer of Zeste (EZH1, EZH2), suppressor of Zeste [SUZ12], and the proteins EED and P55^31–33^. PhoRC contains two proteins, Pho and its homologue Phol, which bind directly to DNA. In mammals, these proteins are encoded by the *Yy1* gene^31^.

Our study identified EED as a core PcG component operating in KNDy neurons of the ARC to prevent the premature initiation of the pubertal process. The results showed that during the early juvenile period, the PcG imposes a repressive histone configuration through an enhanced H3K27me3 content at the 5′ regulatory region of the Kiss1 gene, silencing it’s expression. At the end of juvenile development this inhibition is lifted and replaced with histone modifications associated with active gene expression (H3K9-14Ac and H3K4me3). This configuration results in increased ARC *Kiss1* expression and enhanced GnRH release which leads to the initiation of puberty^27^.

To unveil the role of the PcG complex in the control of puberty we used a gain-of function approach aimed at perturbing the homeostatic make-up of the prepubertal ARC^27^. Prepuberal rats received an intra ARC stereotaxic injection of lentiviral particles carrying either an EED-overexpression vector or a control (GFP) vector. Arcuate-median eminence (ARC-ME) tissue explants were recovered after a week for in vitro incubation and determination of GnRH release in serial samples^27^. This is a robust and reliable method used to determine pulsatile GnRH release from the hypothalamus of small animals^34,35^. EED overexpression in the ARC diminished GnRH pulse frequency without affecting pulse amplitude, ultimately producing a significant reduction of total GnRH output^27^. Moreover, ARC specific EED overexpression delayed pubertal timing and impaired fertility through repression of *Kiss1* repression^27^. These results suggest that before puberty, EED represses KNDy neuronal function by facilitating accumulation of the repressive H3K27me3 mark around the *Kiss1* promoter region, ultimately causing inhibition of the pulse generator.

Here we performed a secondary analysis of these animals to identify the gene networks that may be affected by EED, and that—expressed in either KNDy neurons and/or associated neuronal circuitries—contribute to the activity of the pulse generator and the hypothalamic control of puberty. In the present report we address this issue by using a systems biology approach. We first interrogated the MBH of prepubertal female rats overexpressing EED in the ARC using three different, but complementary approaches: massively parallel sequencing, high throughput targeted qPCR, and conventional RT-qPCR. We then analyzed the resulting data using a partial correlation approach to identify those genes highly correlated with GnRH pulse frequency. Computational methods were used to identify and characterize genetic network architectures regulated by the PcG complex, without prior knowledge of the network(s) structure and function. Finally, we used chromatin immunoprecipitation assays and in vitro approaches to experimentally assess the validity of in silico predictions and gain insight into the biological significance of the changes in network structure caused by EED-driven perturbation.

## Results

### Weighted gene co-expression network analysis reveals a relationship between *Eed*, *Kdm6b and Kiss1* expression

**Figure 1:**
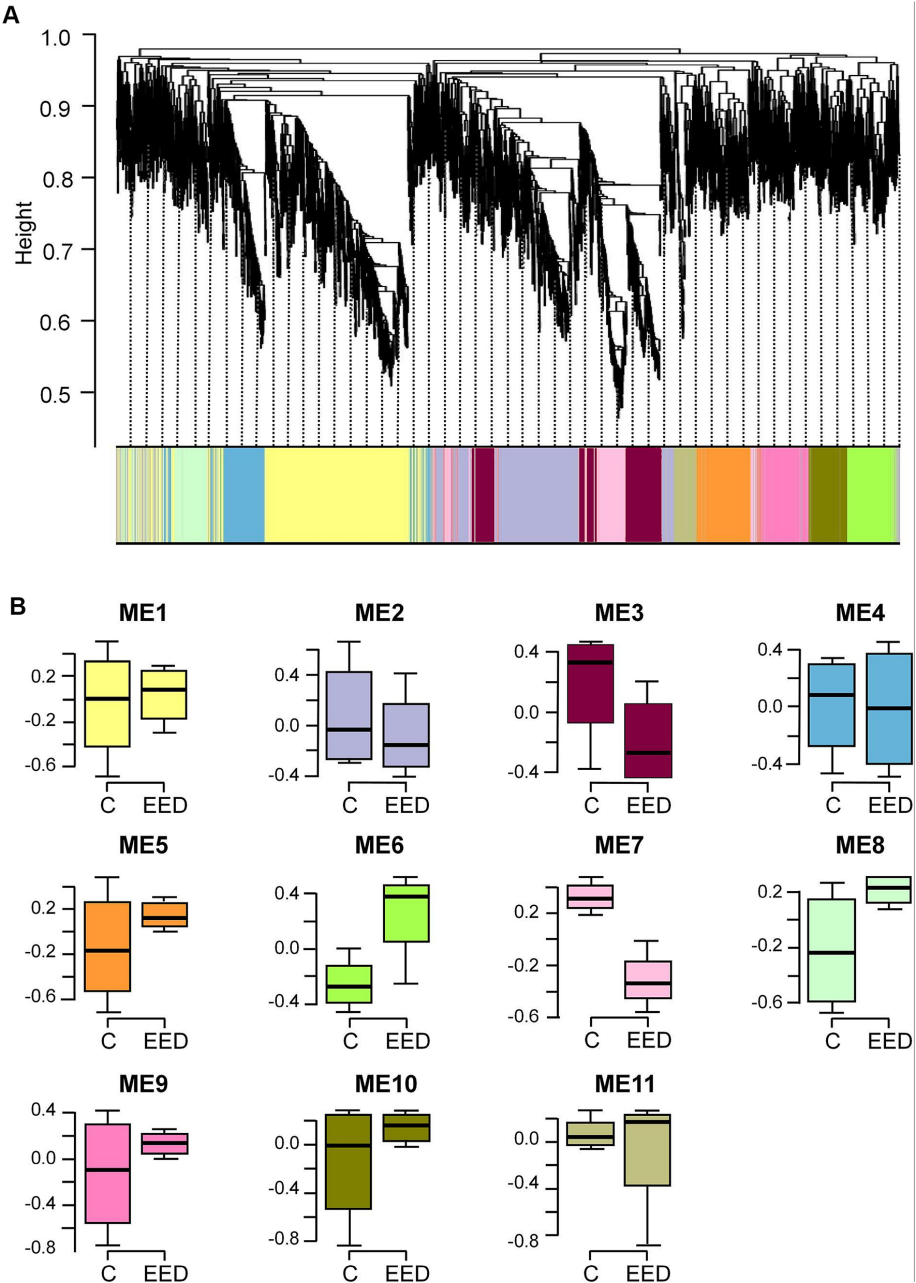
WGCNA analysis of gene expression after EED overexpression targeted to the ARC of prepubertal female rats. (**A**) Dendrogram of WGCNA module memberships of the top 5000 most variable genes detected by RNA-seq in the MBH of prepubertal female rats overexpressing EED in the ARC region of the MBH. The different colors depict the 11 identified modules. (**B**) Boxplots of module eigengene values for control ((**C**), LV-GFP-injected animals) and EED-overexpressing (EED, LV-EED-injected animals) groups (excluding module zero). Boxplot colors map to the dendrogram shown in (**A**).

To discover potential co-expression modules of genes whose expression was altered by elevated levels of EED in the ARC, we subjected the RNA-seq data to Weighted Gene Co-Expression Network Analysis (WGCNA)^36^. For this analysis we utilized the log2 counts per million (CPM) per sample of the top 5000 most variable genes having a minimum average CPM of 1 or > 1, as summarized by the voom function^37^ of the edgeR package^38^ (http://www.R-project.org). The clustering pattern of these 5000 genes is illustrated in Fig. 1A. We also examined alterations in expression pattern as illustrated by the eigengenes of each of the 11 resulting WGCNA-modules (Fig. 1B), and utilized DAVID analysis^39,40^ to determine if genes contained in modules with altered expression in the ARC of *Eed*-overexpressing animals also showed functional overrepresentation.

Only modules 6 and 7 exhibited clear separation of overall expression between LV-EED and LV-GFP-injected animals, with module 6 upregulated and module 7 downregulated in the MBH of rats injected with LV-EED in the ARC. Functional overrepresentation analysis indicated that module 6 was strongly enriched for terms related to signaling, immune response and myelination pathways, while module 7 showed relatively little functional enrichment (Supplementary Table S1A,B). However, several individual members of these co-expression modules were genes that could be important for the epigenetic regulation of pubertal timing. For instance, module 6 (which, as expected, contains *Eed*), also includes *Kat2b*, a gene that encodes an acetyltransferase shown to stabilize the PRC2 complex of PcG^41^ and *Mbd4*, which encodes a protein that recognizes and binds methylated DNA^42^. Module 7, on the other hand, contains *Kiss1* and *Kdm6b* (Supplementary Table S2)*,* an intriguing co-localization because the major function of KDM6B is the demethylation of H3K27me3 at promoter regions targeted by the PcG complex^43,44^. From a mechanistic perspective, we found the hypothesis that KDM6B could regulate *Kiss1* expression by acting as an antagonist of PcG-dependent gene silencing compelling in light of our previous results showing that EED directly represses *Kiss1* activity in KNDy neurons of the ARC^27^. The changes in expression for these and several other genes observed after EED overexpression were all nominally significant (Supplementary Table S2).

### EED decreases glutamatergic gene expression

We noted several genes encoding glutamatergic receptors that were down-regulated by EED overexpression (Supplementary Table S2), including clusters of these genes associated with WGCNA modules 2 and 3. This suggested that an important outcome of enhanced PcG repression is inhibition of glutamatergic transmission. To further understand the potential interplay of *Eed* and *Kdm6b* with genes encoding glutamatergic receptors, in addition to other genes of interest, we used an OpenArray platform to assay a targeted set of 224 genes with potential involvement in the regulation of puberty^45^. In addition, we employed targeted qPCR to assess the expression of genes that were not represented on the OpenArray set or that were not accurately assayed in this platform due to either low levels of expression or imprecise replication.

This study demonstrated significant differential expression of several genes in the MBH of *Eed*-overexpressing animals as compared with the MBH of controls injected with LV-GFP (Supplementary Table S3). In agreement with the RNA-seq results, *Kdm6b* was downregulated in *Eed*-overexpressing animals. In addition, several genes encoding either glutamatergic receptors or molecules involved in glutamatergic transmission were heavily downregulated (Supplementary Table S3). Notably, *Nell2*, a gene selectively expressed in glutamatergic neurons and encoding a glycoprotein that promotes neuronal growth and supports glutamatergic signaling^46^ was the single most downregulated gene among those assayed using the combination of Open Arrays and qPCR.

### *Kdm6b* expression is related to increased GnRH pulse frequency

**Figure 2:**
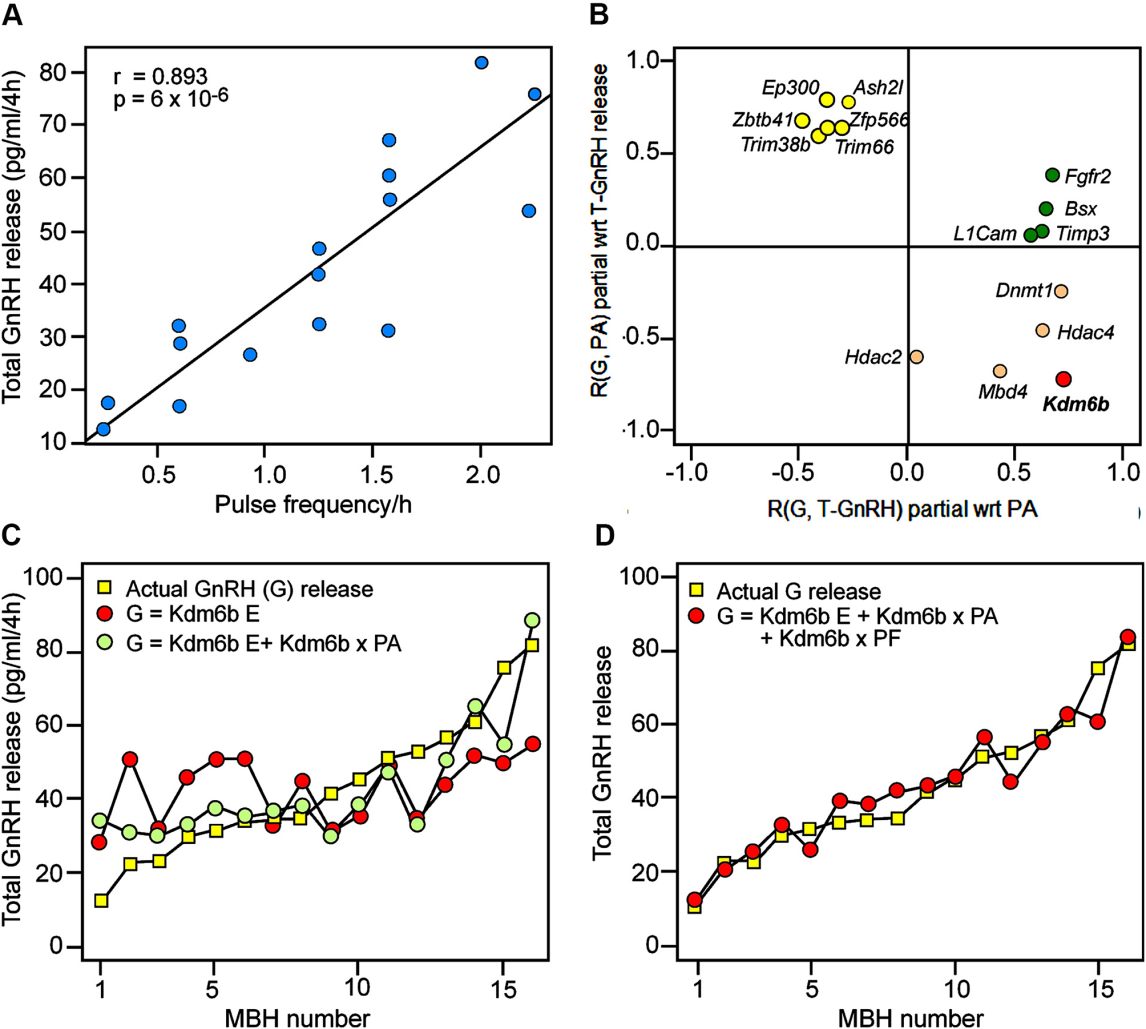
Correlations between gene expression and GnRH release from the MBH after EED overexpression. (**A**) Scatterplot of total GnRH release vs. average GnRH pulse frequency/hour over a 4 h incubation period of MBH fragments derived from late juvenile 28-day-old female rats injected 6 days earlier with a lentiviral expressing GFP alone (n = 8) or EED plus GFP (n = 8). Best-fit linear correlation is indicated by black line. (**B**) Plot of partial correlations (R) of gene expression (G) as assayed by OpenArray/qPCR with total (T) GnRH release and pulse amplitude (PA) with regard to (wrt) other physiological metric. (**C**) Plot of total GnRH release ordered from lowest to highest (yellow squares) and regression model predictions for Ŷ_Total Expression_ = β_intercept_ + β_*Kdm6b* expression_ (red circles) and Ŷ_Total Expression_ = β_intercept_ + β_*Kdm6b* expression_ + β_*Kdm6b* expression x Pulse Amplitude(PA)_ (green circles). MBH number refers to one of the 16 MBH incubated in vitro. (**D**) Plot of total GnRH release ordered from lowest to highest (yellow squares) and regression model prediction for Ŷ_Total Expression_ = β_intercept_ + β_*Kdm6b* expression_ + β_*Kdm6b* expression × Pulse Amplitude (PA)_ + β_*Kdm6b* expression × Pulse Amplitude (PA) × Pulse Frequency(PF)_ (red circles).

To identify genes mostly correlated with GnRH pulsatile release, we first analyzed the basic characteristics of pulsatile GnRH release from incubated ARC-ME fragments and observed that the frequency of GnRH pulsatility correlated strongly with total GnRH release (Fig. 2A), but not with the average amplitude of pulses. We then used a partial correlation analysis strategy to assess the existence of potential relationships between gene expression and GnRH secretion, and found that expression of several genes had a strong partial correlation with either total GnRH release or pulse amplitude when the influence of the other variable was removed (Fig. 2B, Supplementary Table S4). Of these genes, only *Kdm6b* showed a strong positive partial correlation with GnRH release after removal of pulse amplitude correlation as a variable, and a strong negative partial correlation with pulse amplitude, once the effect of correlation with total GnRH release was removed (Fig. 2B). Thus, higher *Kdm6b* expression appears to correlate with more frequent GnRH pulses, consistent with the strong correlation of pulse frequency with overall release noted earlier. This inference was supported by regression analysis; while *Kdm6b* expression on its own was a relatively poor predictor of total GnRH release, addition of a *Kdm6b* expression-pulse amplitude interaction term considerably improved the fit of the regression (Fig. 2C). An additional *Kdm6b* expression-pulse frequency term led to an even tighter fit to total GnRH release (Fig. 2D). Overall, these results suggest that *Kdm6b* plays a significant role in the regulation of GnRH release, possibly via positive control of *Kiss1* expression as suggested by our RNA-seq and Open Array results. However, it is also clear that regulation of *Kiss1* expression alone might not fully account for the alterations in GnRH release we observed. In particular, the partial correlations of *Kdm6b* with GnRH pulse frequency suggested that *Kdm6b* might be involved in regulating additional neuronal excitatory systems controlling GnRH release. The loss of glutamatergic receptor gene expression revealed by our RNA-seq and Open Array analyses (Supplementary Tables S2, S3) supports this assumption.

### PcG/KDM6B are linked to *Kiss1*/glutamatergic/potassium channel gene expression

**Figure 3:**
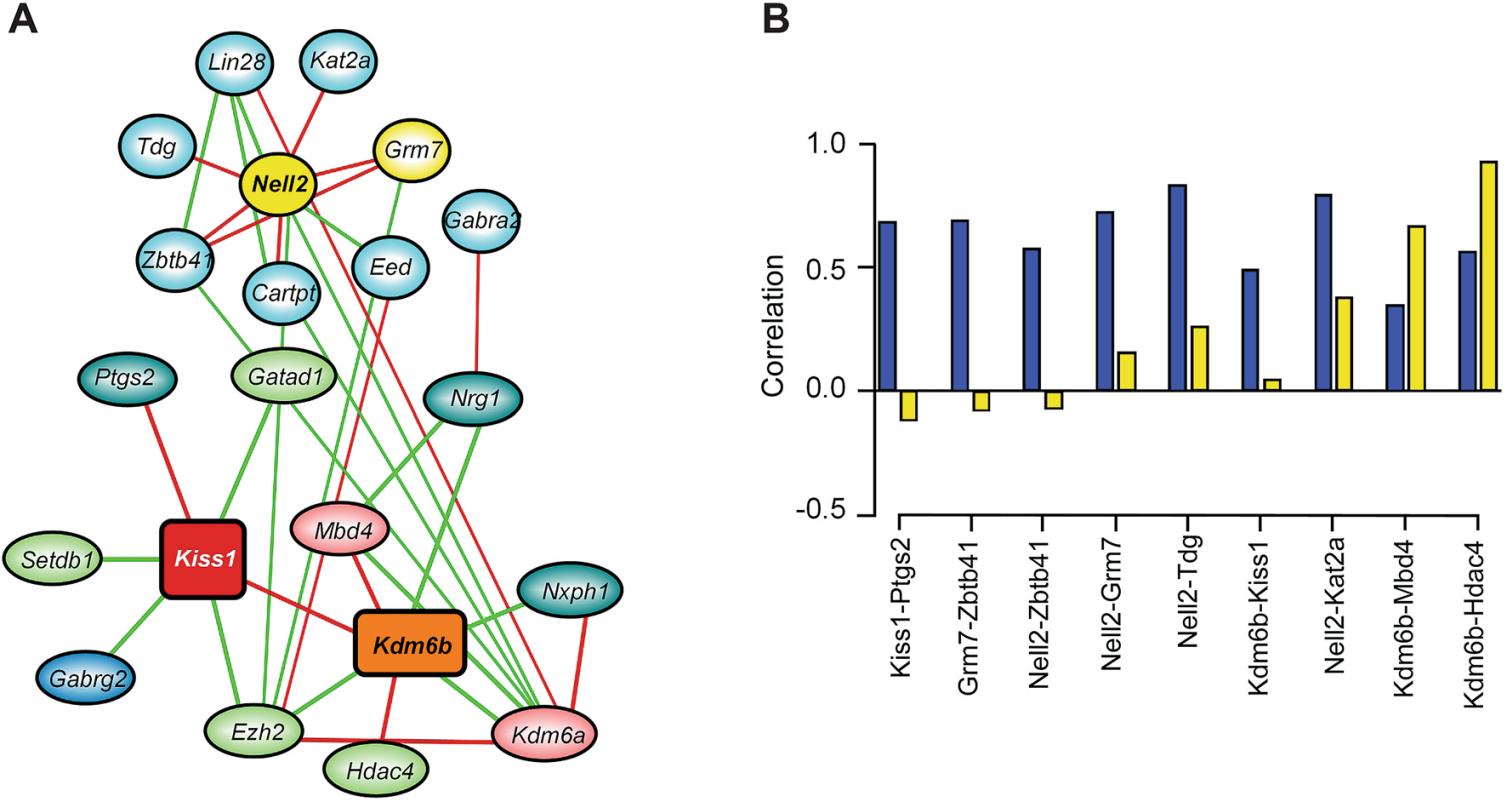
Gene co-expression networks in the MBH of immature female rats injected with either LV-GFP or LV-EED in the ARC. (**A**) First and second-neighbor network of strong co-expression edges for *Kiss1* and *Kdm6b*. Positively correlated edges are red, negatively correlated edges are green. First neighbors involved in primarily negative epigenomic regulation are indicated in light green ovals, while positive epigenomic regulators are indicated in red ovals. Non-epigenetic genes are blue ovals. Glutamatergic-related genes are indicated in yellow ovals, other in cyan ovals. (**B**) Histogram depicting expression correlations of pairs of genes involved in positively correlated strong co-expression relationships with *Kdm6b, Kiss1, Nell2* and *Grm7* under control conditions (blue bars) and after EED overexpression (yellow bars).

In addition to the above described expression analysis, we performed a compressive sensing-based co-expression network inference on the OpenArray data and targeted RT-PCR data as described earlier^45^. We utilized this method instead of WGCNA due to the smaller number of genes examined by our PCR analyses and the ability of the compressive sensing-based method to detect robust relationships between individual genes. The results of this analysis indicate a strong direct positive relationship between *Kiss1* and *Kdm6b* expression (Fig. 3A) that recapitulates the clustering of these two genes in module 7 of our WGCNA analysis. Importantly, both genes also showed a robust negative correlation with *Ezh2*, the catalytic member of the PRC2 H3K27-methyltransferase complex^28^, supporting the hypothesis of an antagonistic role for KDM6B and the PcG complex^22^ in the regulation of *Kiss1* expression. In addition, *Kiss1* showed a negative relationship with *Gatad1* (previously shown to repress puberty^45^), *Setdb1*, which encodes an H3K9-methyltransferase that catalyzes the synthesis of H3K9me3, a repressive histone mark^47,48^, and *Gabrag2*, the gene encoding the gamma2 subunit of a GABA_A_ receptor. This last observation is interesting, because alterations in GABAergic/glutamatergic signaling balance could be a factor underlying the relationship of pulse frequency and overall GnRH release we observed in our physiological experiments. Intrigued by this possibility, we searched for the second neighbors of both *Kiss1* and *Kdm6b* in the co-expression network to identify additional genes of interest.

We found that another GABA receptor gene, *Gabra2,* is a second neighbor of *Kdm6b* and that two genes encoding proteins involved in glutamatergic transmission*, Nell2* and *Grm7,* are second neighbors to both *Kdm6b* and *Kiss1* (Fig. 3A). *Grm7* encodes a Group III glutamatergic metabotropic receptor involved in the etiology of mood disorders^49^. Interestingly, *Nell2* and *Grm7* are direct neighbors of *Ezh2*, suggesting the likelihood of a regulatory relationship between *Kdm6b,* the PRC2 complex, and genes encoding glutamatergic receptors. Further analysis of these associations revealed that the majority of positively correlated co-expression edges between the highly-connected *Kdm6b, Kiss1, Nell2 and Grm7* nodes and neighboring genes observed in the MBH of LV-GFP injected animals diminished considerably in strength after EED overexpression (Fig. 3B). The exceptions were the positive correlations between *Kdm6b* and *Mbd4*/*Hdac4* expression as the strength of these associations was not reduced by EED overexpression. Altogether, these results suggest that the PcG complex keeps puberty in check not only by repressing *Kiss1* expression, but also by disrupting co-expression of functionally associated genes that interact with *Kiss1* within the boundaries of a gene network involved in excitatory neurotransmission.

In light of these findings, we re-examined the co-expression modules from our RNA-seq experiment and discovered that a number of glutamatergic genes showing at least nominally significant downregulation under EED-overexpression are components of modules 2 and 3 (Supplementary Table S1C,D). In addition, we noted a highly significant enrichment for potassium channel genes in these modules (Supplementary Table S1C,D). Strikingly, some of the potassium channel genes that were downregulated have been characterized as leak channels involved in maintaining neuronal membrane potentials near action potential thresholds (e.g. *Kcnk4*, *Kcnk5*)^50^ or with speeding neuronal recovery after action potentials through delayed rectification (e.g., *Kcna1*)^51^. Additionally, *Kcnn1*, a calcium-responsive potassium channel involved in suppression of membrane excitability and regulation of spike train intervals^52^ is located in the upregulated module 6, consistent with its oppositional role in regulating membrane potential compared to the majority of potassium channel genes located in downregulated modules.

Because many of these genes were not present in the OpenArray design we could not directly assess the existence of relationships with *Kdm6b* or members of the PcG complex. To overcome this limitation, we used the GeneMANIA database of known relationships between genes^53^ and found strong co-expression, genetic interaction and pathway interconnection between glutamatergic and potassium channel genes downregulated by EED overexpression, including *Nell2* and *Grm7* (Supplementary Fig. S1). The concordance of this finding with the basic structure of our inferred strong co-expression network and WGCNA modules suggest that the genes most prominently co-regulated with *Kiss1* by EED/KDM6B belong to a cohort of genes involved in excitatory neurotransmission.

### Targeted RT-PCR confirms the in vivo network differential co-expression predictions

**Figure 4:**
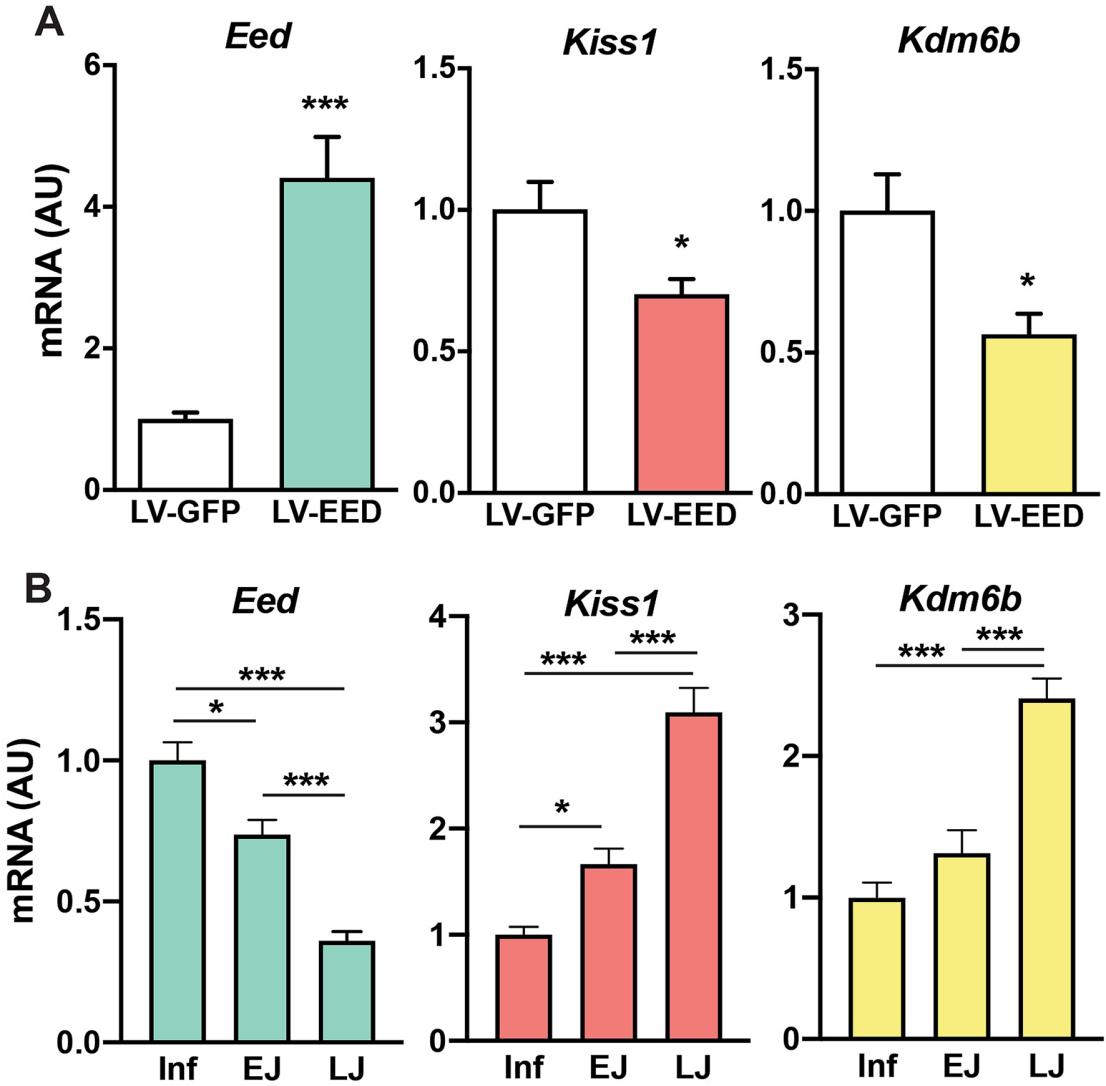
Changes in MBH gene expression elicited by EED overexpression or during prepubertal development. (**A**) The animals received a bilateral injection of LV-GFP or LV-EED in the ARC at the beginning of juvenile development (22-days of age), and the MBH was collected on postnatal day 28; mRNA levels were measured by qPCR. Results are expressed as fold change with respect to control values. *p < 0.05, ***p < 0.001 vs. LV-GFP- Control treated rats. (Student’s t-test) (n = 8 per group). (**B**) Rats were euthanized at 3 different stages: Inf; infantile (14 days of age), EJ: early juvenile (21 days of age) and LJ: late juvenile (28d of age) of prepubertal development. MBH was collected to determine mRNA levels by qPCR. Results are expressed as fold change with respect to Inf values. *p < 0.05, ***p < 0.001, One Way ANOVA followed by Student–Newman–Keuls Test (n = 6 per group).

To confirm the EED-induced changes in expression predicted by both WCGNA analysis of RNA-seq data and our compressive sensing-based co-expression network inference we used a targeted RT-PCR approach. Hypothalamic *Eed* overexpression significantly decreased *Kiss1* and *Kdm6b* mRNA (Fig. 4A), confirming the RNAseq data. Additionally, we measured a subset of mRNAs encoding the glutamatergic receptors and potassium channels identified in Supplementary Fig. S1. While some genes (*Kat2b, Kcne2* and *Kcnn1*) where moderately activated by LV-EED, most of them showed a significant reduction in mRNA expression (Supplementary Fig. S2). These results indicate that genes other than *Kiss1,* like those encoding a defined subset of glutamatergic receptors and potassium channels, are also under PcG repressive control in the MBH in vivo.

To further characterize our gene co-expression predictions, we studied selected gene expression profiles in the MBH during normal prepuberal development. Corroborating our earlier findings^27^, we observed that *Eed* expression is high during the infantile period (Inf: PND14), and decreases significantly during the early juvenile (EJ: PND21) to late juvenile (LJ: PND28) transition. Concomitantly, *Kiss1* and *Kdm6b* expression is enhanced, further reinforcing the possibility that EED functions as a repressor of both *Kiss1* and *Kdm6b* expression in the hypothalamus (Fig. 4B).

### EED represses gene expression in vitro trough increased H3K27me3 abundance at the promoters of network genes

**Figure 5:**
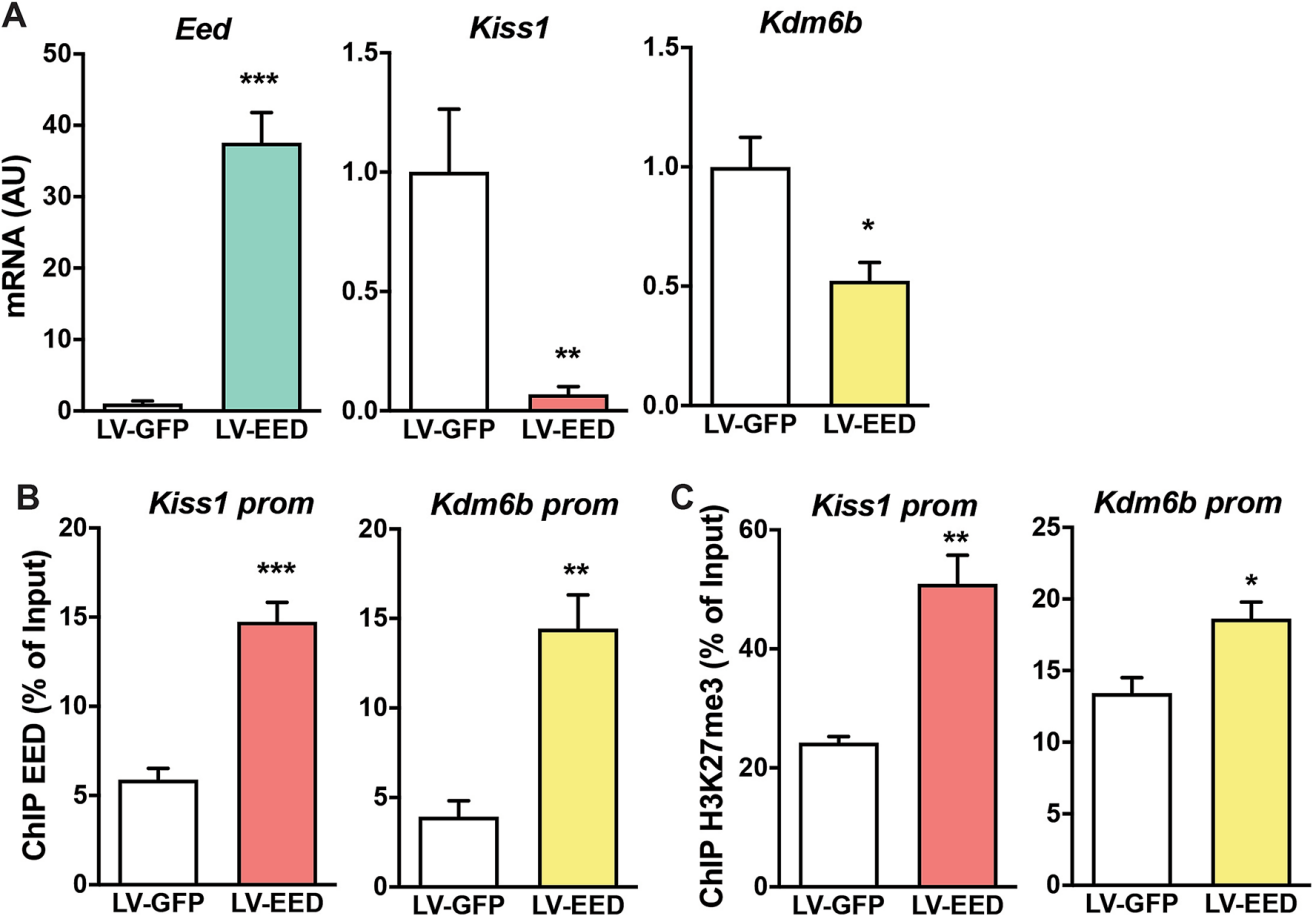
Changes in Gene Expression and recruitment of EED and H3K27me3 to the promoter of network genes. (**A**) Gene expression in R22 cells stably expressing EED. mRNA levels were measured by qPCR. Results are expressed as fold change with respect to control LV-GFP group. *p < 0.05, ***p < 0.001 vs. LV-GFP- Control (n = 4 per group). (**B**) Recruitment of EED to the promoters of *Kiss1* and *Kdm6b* in R22 cells overexpressing EED. (**C**) Increased H3K27me3 abundance at the promoter of *Kiss1* and *Kdm6b* in R22 cells after EED overexpression. Results are expressed as fold-change with respect to cells transduced with LV-GFP. *p < 0.05, **p < 0.01, ***p < 0.001 vs. LV-GFP treated cells (Student’s t-test) (n = 4 per group).

We next compared the expression profiles of genes in the MBH of control and EED-overexpressing animals in vivo with profiles observed in hypothalamic R22 cells overexpressing EED in vitro. With exception of *Kcnn1* whose mRNA levels increased under EED overexpression in vivo*,* but decreased in EED overexpressing cells in vitro, the changes of expression induced by EED were similar in vivo and in vitro for all other genes analyzed (Fig. 5A, Supplementary Fig. S3). Surprisingly, two glutamatergic markers, *Nell2* and *Grm7,* are strongly expressed in the MBH, but not in R22 cells (Supplementary Fig. S3). Despite this discrepancy, these results as a whole support the use of R22 cells as a valid in vitro system to study hypothalamic genetic interactions.

While our physiological and co-expression analyses strongly indicate that EED*/*PRC2 represses the activity of genes important for pubertal development, they do not inform us as to whether or not EED is directly recruited to the regulatory regions of these genes. To address this question, we used a chromatin immunoprecipitation approach, utilizing R22 cells stably overexpressing EED.

We first determined if EED is recruited to the promoter regions of *Kiss1* and *Kdm6b* (Fig. 5B)*,* as well as to the promoters of glutamatergic and potassium channel genes previously identified as being EED targets (Supplementary Fig. S1). We then assessed H3K27me3 levels at the promoters, as a proxy for *Eed*/*Kdm6b* balance. Recruitment of EED to the *Kiss1* and *Kdm6b* promoters increased significantly after EED overexpression (Fig. 5B), as well as in glutamatergic and potassium channel genes (Supplementary Fig. S4A). The content of H3K27me3 also increased (Fig. 5C, Supplementary Fig. S4B), indicating that—consistent with its role in PRC2 function^28^—EED facilitates the deposition of H3K27me3 at the promoter region of downstream target genes. Overall, these results support our gene network prediction and the notion that the PRC2 complex down-regulates the promoters of not only *Kiss1* and *Kdm6b*, but of several glutamatergic-related and potassium channel genes, in a manner consistent with its previously demonstrated role as a negative modulator of pubertal timing^27^. Notably, the majority of the potassium channel genes repressed by EED have been shown to be involved in maintaining membrane potential or facilitating recovery after the action potential^50–52^. This suggests that their regulation, along with regulation of glutamatergic genes, represents a physiological mechanism underlying the antagonistic relationship of *Eed* and *Kdm6b* expression with the loss of GnRH pulsatility that occurs in the presence of elevated EED levels in the ARC of animals approaching puberty.

### KDM6B counteracts EED-mediated effects on selected gene network members

From the above-mentioned in vivo and in vitro results, it became clear that by interacting with promoter regions and increasing H3K27me3, EED downregulates the expression of *Kdm6b, Kiss1* and other second tier genes involved in glutamate signaling and potassium dependent membrane transporters. To determine if these genes are directly affected by EED or by the loss in *Kdm6b* expression, we assessed the ability of *Kdm6b* to counteract the repressive activity of EED on a selected group of genes.

**Figure 6:**
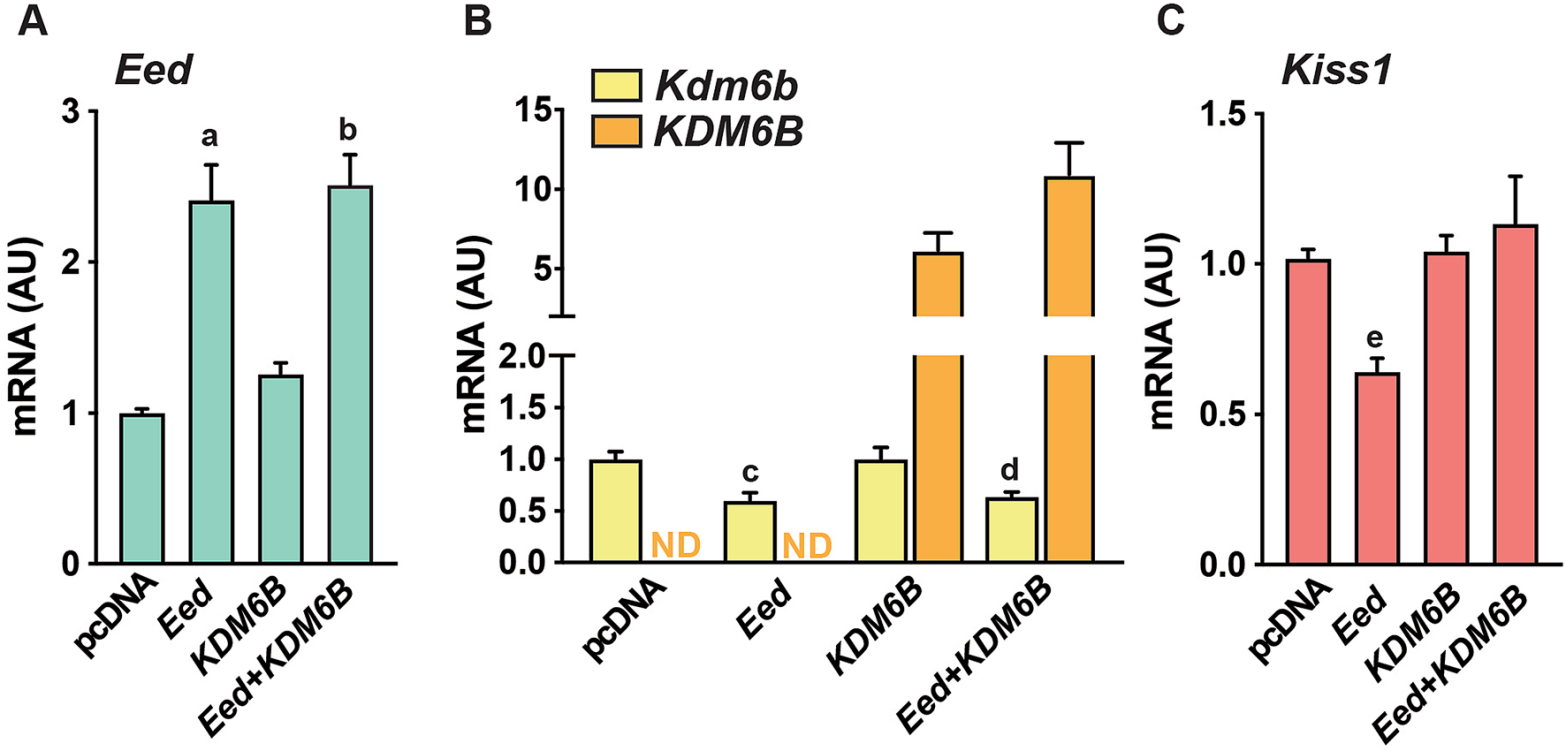
KDM6B counteracts the effects of EED. Rat R22 cells were transiently transfected with rat *Eed*, a human *KDM6B* expression vector or both. Expression of (**A**) rat *Eed*, (**B**) rat endogenous *Kdm6b (yellow bars)* and human exogenous *KDM6B* (orange bars) and, (**C**) rat *Kiss1* was determined 48 h after transfection. mRNA levels were measured by qPCR. Results are expressed as fold change with respect to control-pcDNA values. a,b p < 0.001 vs pcDNA and *KDM6B* groups. c, d, p < 0.05 vs pcDNA and *KDM6B* groups. e, p < 0.05 all other groups (One Way ANOVA followed by Student-Newman–Keuls test) (n = 3 per group). N.D. = not detectable.

We observed that transient transfection of the rat *Eed* overexpression construct into R22 cells lead to the expected increase in *Eed* expression (Fig. 6A), while downregulating endogenous *Kdm6b* (Fig. 6B, yellow bars), in concordance with the in vivo results of Fig. 4. We also observed that overexpression of human *KDM6B* (Fig. 6B, orange bars), antagonizes the repressive effect of *Eed* on endogenous *Kiss1* mRNA expression (Fig. 6C), and partially, but significantly, counteracted the effect of *Eed* on *Grik5, Kcnk4* and *Kcnk5* (Supplementary Fig. S5). As indicated earlier these three genes are involved in excitatory neurotransmission and are co-regulated with *Kiss1* by the EED/KDM6B complex (Fig. 3A).

Altogether, these results lend credence to the concept that the Polycomb complex keeps puberty in check by repressing the *Kiss1* gene directly, and indirectly through repression of *Kdm6b,* a histone demethylase that counteracts the effect of EED on chromatin structure. Moreover, the Polycomb complex also controls a group of second-tier genes involved in the neuroexcitatory control of puberty.

## Discussion

In mammals, pulsatile GnRH secretion is a mode of neurosecretion characterized by the periodic release (every 30 min) of discrete amounts of GnRH into the portal circulation connecting the hypothalamus to the pituitary gland^54,55^. Increased GnRH pulse frequency is crucial for female reproductive function as it is required for both pubertal maturation and the follicular and preovulatory phases of the menstrual cycle in adults^56,57^. These episodes of GnRH release closely follow bursts of multi-unit electrical activity in the ARC^55,58–60^, and require the coordinated action of the neuropeptides neurokinin B/kisspeptin and dynorphin^61,62^ to occur. Although some of the genetic networks coordinating GnRH neuronal activity have been identified^3,63^, little is known about the epigenetic mechanisms that may coordinate gene networks involved in facilitating GnRH pulsatile release during pubertal development. The present report addresses this issue.

We have previously demonstrated that a Polycomb group-dependent epigenetic mechanism of transcriptional repression operates within the ARC nucleus of the hypothalamus to time the initiation of female puberty. This repressive influence leads to diminished pulsatile GnRH release, and involves transcriptional inhibition of the ARC *Kiss1* gene, which is crucial for GnRH release^8^. Peripubertal female rats overexpressing *Eed (*a component of the Polycomb Repressive Complex 2) in the ARC showed decreased *Kiss1* expression, increased GnRH inter-pulse interval, decreased total GnRH release and delayed puberty^27^. Thus, when EED abundance in the ARC increases GnRH secretion decreases and puberty is delayed. This in vivo intervention was performed in the early juvenile to late juvenile period of pubertal development (PND21–PND28), without affecting the AVPV kisspeptin population. Although AVPV Kisspeptin neuros are also involved in the pubertal process, mainly by contributing to the preovulatory surge of gonadotropins^64–67^, we focused our analysis on the ARC, home of KNDy neurons, believed to represent a core component of the GnRH pulse generator.

The present studies provide insights into the integrative mechanisms underlying the epigenetic regulation of puberty by identifying a role of KDM6B, a histone demethylating enzyme, in the control of pulsatile GnRH release before the acquisition of reproductive maturity. The Polycomb complex catalyzes trimethylation of histone 3 at lysine 27 (H3K27me3), and use this histone modification to repress gene expression^43,68^. In turn, KDM6B regulates this process by erasing the K27 methylation mark from H3, and thus reducing the prevalence of H3K27me3 at gene regulatory regions^44^.

Using a gain of function and a system biology approach we discovered the existence of a genetic network in the ARC that displays *Kdm6b* as a central node, with *Kiss1* and several epigenetic related genes (*Ezh2, Hdac4, Kdm6a, Mbd4*) as first neighbors. Our results also demonstrated that *Kdm6b* not only regulates the expression of *Kiss1*, but also the transcriptional activity of a cohort of genes involved in excitatory neurotransmission, and therefore in the stimulatory neural control of GnRH release^39–41^. These genes encode glutamatergic receptors (*Grm7, Nell2*, *Grik5, Grin2a/d, Grin1* and *Gria1*), a glutamate release-inducing molecule (*Nell2*), and potassium channels (*Kcnh3*, *Kcnc3, Kcnk4/5*) responsive to arachidonic acid metabolites^42^ and present in the hypothalamus^43^, where they facilitate neuronal excitability.

A remarkable feature of this KDM6B-dependent regulatory system is the strong correlation that exists between *Kdm6b* expression and GnRH pulse frequency, as revealed by the correlation analysis of the 5000 most variable genes in the ARC of control vs *Eed* overexpressing animals. Functional analysis of *Eed/Kdm6B* interactions revealed that EED inhibits gene expression by recruiting the repressive H3K27me3 histone mark to gene promoters expressed in the ARC. When *Kdm6b* content is enhanced, trimethylation of H3K27 is diminished and the repressive effect of EED on *Kiss1* and the network genes involved in excitatory neurotransmission is lost. Our bioinformatic approach suggests that the PcG complex is a central node in the repressive control of ARC *Kiss1, Kdm6b,* glutamate receptors and potassium channels expression. As puberty approaches, waning *Eed* inhibition leads to increased *Kdm6b* expression which, in turn, reverses the chromatin structure of repressed genes, facilitating gene expression. This makes *Kdm6b* a second-tier regulatory gene of *Kiss1*, an effector gene. These results are, therefore, consistent with the view that *Kdm6b* functions in a highly dynamic manner to facilitate the rhythmic activity of GnRH neurons during pubertal development, and controls GnRH pulse frequency by antagonizing the repressive effect of the PcG complex on genes involved in the stimulatory control of GnRH secretion.

ARC activity is controlled by many other regulatory signals including growth factors and hormones, like leptin and insulin which reach the ARC directly though fenestrated blood vessels of the median eminence of the hypothalamus^69–73^. These signals provide growth and metabolic status information into the neuronal networks that control food intake and reproductive development. On the other hand, it was recently demonstrated that ARC neurons are indirectly controlled trough leptin-receptor containing neurons present in the premammillary nucleus, affecting pubertal development^74^. These leptin sensitive neurons project and make contacts with GnRH and KNDy neurons at the level of the ARC and median eminence, possibly affecting their activity through glutamate release^74^. Activation of growth factor and hormone receptors induce increased activity of several intracellular pathways including the phosphatidylinositol-3-kinase (PI3K)/AKT/mammalian target of rapamycin (mTOR), JAK/STAT and the MAPK pathways^75–80^. While the activation of the PI3K/AKT and MAPK pathways phosphorylate several PcG members, causing their dissociation from target regulatory regions^81,82^, the activation of PI3K/AKT and STAT pathways induce the phosphorylation of KDM6B^83^ and increased K27-dependent demethylase activity. Since our gene network results identifies not only ARC Kiss1 but also several glutamate receptors as targets of the EED/KDM6B system, we can hypothesize that increased developmental or metabolically driven activation of several kinase pathways controlling ARC function converge into an EED/KDM6B centered transcriptional/epigenetic pathway that regulates pubertal development. We have previously demonstrated the participation of the NAD+ dependent protein deacetylase SIRT1 in a metabolically regulated repressive complex that, together with the PcG family controls ARC *Kiss1* expression and pubertal development^84^. It appears that an EED/KDM6b regulatory system plays an important role not only in the hypothalamic control of puberty but also in the control of other critical development events including the first mammalian cell lineage commitment^85^, and mammalian embryo implantation^86^.

By demonstrating the involvement of the EED/KDM6b system in the control of GnRH output at puberty, our findings provide a compelling example of a critical developmental event in the lifespan of an individual subject to tight epigenetic control.

## Methods

### Animals

The animals used were those described in a previous publication^27^. They were juvenile (14–28 days of age) Sprague Dawley female rats, and were obtained from Charles River Laboratories international, Inc. (Hollister, CA). Upon arrival, the rats were housed in a room with controlled photoperiod (12/12 h light/dark cycle) and temperature (23–25 °C), with ad libitum access to tap water and pelleted rat chow. The use of rats was approved by the ONPRC Institutional Animal Care and Use Committee (IACUC) in accordance with the NIH guidelines for the use of animals in research. All the studies where carried out in compliance with the ARRIVE guidelines.

### Tissue collection during the prepuberal phase

To determine the changes in hypothalamic gene expression during female prepubertal phases, rats were euthanized at 3 different stages: Inf; infantile (14 days of age), EJ: early juvenile (21 days of age) and LJ: late juvenile (28 days of age). According to criteria previously established by us^1,87^, 21-days-old animals are considered to be in the juvenile phase of prepubertal development. At this time, the vagina is not yet patent and the uterine weight is 60 mg or less, with no accumulation of intrauterine fluid. At 28 days of age, the rats are in the late juvenile (LJ) phase of prepubertal development; their vagina is closed and there are no signs of intrauterine fluid accumulation. Animals in this phase exhibit a diurnal change in pulsatile plasma LH levels, with the LH pulses becoming more pronounced in the afternoons^88^. Like in humans and monkeys, this is the first hormonal manifestation of the increase in central drive that initiates puberty^89^. All animals were euthanized between 1600 and 1700 h, by CO2 exposure at 5 L/min in a regular rat filter top cage. One minute after breathing stopped, confirmation of euthanasia was performed by decapitation in accordance to the American Veterinary Medical Association guidelines. The MBH was immediately dissected and frozen on dry ice, as previously described^27^.

### Preparation of EED-expressing lentiviral particles

The generation of lentiviral particles over-expressing the rat *Eed* gene (LV-EED) was reported earlier^27^. Shortly, a 3rd generation vector system was used. The promoter sequences of the 5′-LTR were replaced by the cytomegalovirus (CMV) promoter producing a heterologous U3 (htU3) promoter^90,91^ followed by the rat insulin II intron A sequence to enhance gene expression^92,93^. The rat *Eed* gene tagged with the human influenza hematogglutinin epitope (HA) was cloned into the BamHI-EcoRV sites, followed by an internal ribosome entry site (IRES) and a cDNA encoding an enhanced green fluorescent protein (GFP).

Lentiviral particles were produced following standard protocols as previously described^27,45,94–96^. Briefly, we co-transfected 293 T cells with 4 plasmids; the LV vector, pLP1, pLP2, and pLPv (Invitrogen) using calcium phosphate. pLP1 expresses *gag* and *pol*; pLP2 expresses *Rev*; and pLPv expresses the VSVG envelope protein. Viral purification and concentration were performed by ultracentrifugation. Virus titer was determined by infection of naive 293 T cells with serial dilutions of the viral stock followed by quantitation of fluorescent cells by flow cytometry. Titer was expressed as transducing units per milliliter (TU/ml).

### Stereotaxic delivery of LV-EED

LV particles carrying an *Eed* transgene were stereotaxically delivered to the arcuate nucleus (ARC) of the hypothalamus of 22-day-old female rats as reported^27^. In short, animals were anesthetized with isoflurane vapor in 100% oxygen and positioned on a stereotaxic instrument (David Kopf Instruments, Tujunga, CA). Bilateral intra-ARC injections of 1 μl LV-GFP or LV-EED was performed with a 10 μl Hamilton micro-syringe using the coordinates: ± 0.3 mm lateral from midline, 0.2 mm anterior from Bregma and 9.6 mm vertical from the surface of the brain as described before^27,45,97,98^. After surgery, animals were given (1 mg/ml; 50 μl/rat) ketoprofen and kept under a warming lamp until fully awake.

### Physiological setup and GnRH release from ARC-median eminence (ME) explants in vitro

The medial basal hypothalamic (MBH) tissue used for the present analysis was that previously reported^27^. The MBH was dissected from 28-day-old female rats that had received six days earlier a bilateral intra-ARC microinjection of a lentiviral (LV) construct expressing rat *Eed* and Green Fluorescent Protein (GFP) under the control of the CMV promoter (LV-EED) (n = 8). Control animals received injections of the same LV expressing only GFP (LV-GFP) (n = 8). The ARC-median eminence (ME) region of each animal was dissected by a rostral cut along the posterior border of the optic chiasm, a caudal cut immediately in front of the mammillary bodies, and two lateral cuts half-way between the medial eminence and the hypothalamic sulci. The thickness of the tissue fragment was about 2 mm and immediately placed in 250 µl Krebs–Ringer’s bicarbonate (KRB) buffer, pH 7.4, containing 4.5 mg/ml d-dextrose at 37 °C under an atmosphere of 95% O2, 5% CO2 with shaking (60 cycles per minute), as described previously^27^. After 30 min of preincubation, the incubation medium was removed every 7.5 min and immediately replaced with 250 µl of fresh medium, this was repeated for 4 h. GnRH released to the medium was determined in 150 µl aliquots. At the end of the incubation, the ARC-ME fragments were collected and processed for isolation of total RNA, which for the purpose of the present study was used for massively parallel sequencing (RNA-seq), quantitative high-throughput PCR (Open Array platform), and targeted RT-qPCR.

For GnRH detection, pure GnRH (Sigma L-7134, St. Louis, MO) was iodinated with I-125. Free I-125 was separated from I-125-GnRH by Sephadex QAE-25 column. GnRH standards were prepared in the same KRB used for the samples in the study. The radioimmunoassay (RIA) has been described in detail before^99^. Final anti-GnRH (EL-14) antibody dilution was 1:144,000. The sensitivity of the assay was 0.02 pg/tube and the range was100 pg/tube with intra- and interassay variation 9% and 12%, respectively.

### Correlation analysis of GnRH release and gene expression

We used a partial correlation approach using the ppcor package in R to determine partial correlations of gene expression as determined by OpenArray with total GnRH release and pulse amplitude after removing the correlation of the other physiological output. The ppcor test function was used to evaluate the significance of each partial correlation, and the significance values were then adjusted using the Benjamini–Hochberg multiple testing correction.

We also performed a regression analysis on the relationship of Kdm6b expression to total GnRH output using a standard linear regression model. Models included the intercept and a Kdm6b expression term, as well as either an interaction term for Kdm6b expression and GnRH pulse amplitude or both Kdm6b/pulse amplitude and Kdm6b/pulse frequency interaction terms.

### EED overexpression in R22 hypothalamic cells

#### Lentivirus infection in vitro

The ability of the LV-EED to alter gene expression and trigger changes in abundance of H3K27me3 at putative target promoters was examined using the immortalized R22 hypothalamic cell line (Cedarlane, Burlington, NC). The cells were plated in DMEM medium at 400,000 cells/well using 12-well plates. Twenty-four hours later, the cells were transduced with the viruses at a multiplicity of infection (MOI) of 5 to 1. Control cells were transfected with LV particles expressing enhanced green fluorescence protein (eGFP) under the control of the CMV promoter and lacking Eed (LV-GFP). Three days after the infection, transduced cells (identified by their expression of eGFP) were isolated by flow cytometry to produce a pure population of cells. These cells were expanded and re-plated onto 12 well plates at a density of 300,000 cells/plate. Three days later, the cells were collected, aliquoted and stored at − 80 °C before extraction of total RNA or chromatin (see below).

#### Nucleofection in vitro transfection

R22 cells were transfected using the Amaxa 4D-Nucleofector Optimization Protocol for primary cells (Lonza, Morristown, NJ). R22 cells were grown in culture plates containing DMEM/High glucose supplemented with 10% FBS in a humidified 37 °C/5% CO_2_ incubator. Cells were harvested by trypsinization and 6 × 10^6^ cell were resuspended in 82 ul of 4D-nucleofector solution with 18 ul of supplement solution and 6ug of four different plasmids: empty pcDNA (control), Eed-pcDNA, pKdm6b-pcDNA and pEed-pcDNA/Kdm6b-pcDNA. Eed-pcDNA was described previously by us^27^ and Kdm6b-pcDNA was obtained from Addgene (Plasmid # 24167). Each mix was transferred to a Nucleocuvette and electroporated using the 4D-nucleofector Core Unit (LONZA) using the program CA-137. After the electroporation procedure, cells were incubated at room temperature for 5 min and then transferred to 10 mm culture dishes and incubated for 48 h in DMEM/High glucose media supplemented with 10% FBS. Transfection efficiency was tested using R22 cells electroporated with pEGFP vector. All transfections where performed at three different times and in triplicate.

### RNA extraction, reverse transcription, and quantitative (q)PCR

Total RNA was extracted from tissues (MBH) and R22 cells using the RNeasy mini kit (Qiagen, Valencia, CA) following the manufacturer’s instructions. RNA concentrations were determined by spectrophotometric trace (Nanodrop, ThermoScientific, Wilmington, DE). Total RNA (2000 ng), was transcribed into cDNA in a volume of 20 μl using 4 U Omniscript reverse transcriptase (Qiagen). To determine the relative abundance of the mRNAs of interest, we used the SYBR GreenER qPCR SuperMix system (Invitrogen, Carlsbad, CA). Primers for PCR amplification (Supplementary Table S5) were designed using the PrimerSelect tool of DNASTAR 14 software (Madison, WI) or the NCBI online Primer-Blast program. PCR reactions were performed in a total volume of 10 μl containing 1 μl of diluted cDNA or a reference cDNA sample (see below), 5 μl of SYBR GreenER qPCR SuperMix and 4 μl of primers mix (1 µM of each gene specific primer). The PCR conditions used were 95 °C for 5 min, followed by 40 cycles of 15 s at 95 °C and 60 s at 60 °C. To confirm the formation of a single SYBR Green-labeled PCR amplicon, the PCR reaction was followed by a three-step melting curve analysis consisting of 15 s at 95 °C, 1 min at 60 °C, ramping up to 95 °C at 0.5 °C/s, detecting every 0.5 s and finishing for 15 s at 95 °C, as recommended by the manufacturer. All qPCR reactions were performed using a QuantStudio 12 K Real-Time PCR system; threshold cycles (CTs) were detected by QuantStudio 12 K Flex software. Relative standard curves were constructed from serial dilutions (1/2 to 1/500) of a pool of cDNAs generated by mixing equal amounts of cDNA from each sample. The CTs from each sample were referred to the relative standard curve to estimate the mRNA content/sample; the values obtained were normalized for procedural losses using glyceraldehyde-3-phosphate dehydrogenase (*GAPDH*) mRNA or peptidylprolyl isomerase A (Ppia) as the normalizing unit.

### Massively parallel RNA sequencing (RNA-seq)

Total RNA from ARC-ME fragments derived from rats receiving LV-EED or LV-GFP particles into the ARC and incubated in vitro to examine changes in pulsatile GnRH release was subjected to RNA-seq. The RNA-seq procedure was carried out by the OHSU Massively Parallel Sequencing Shared Resource. RNA-seq libraries were prepared using the TruSeq Stranded protocol with ribosomal reduction (Illumina, San Diego, CA). Briefly, 600 ng of total RNA per sample were depleted of ribosomal RNA using RiboZero capture probes (Illumina). The purified RNA was then fragmented using divalent cations and heat, and the fragmented RNA was used as template for reverse transcription using random hexamer primers. The resulting cDNAs were enzymatically treated to blunt the ends, and a single “A” nucleotide was added to the 3′ ends to facilitate adaptor ligation. Standard six-base pair Illumina adaptors were ligated to the cDNAs and the resulting DNA was amplified by 12 rounds of PCR. All of the above procedures were carried out following the protocol provided by Illumina. Unincorporated material was removed using AMPure XP beads (BeckmanCoulter, Brea, CA). Libraries were profiled on a Bioanalyzer instrument (Agilent, Santa Clara, CA) to verify: (a) the distribution of DNA sizes in the library, and (b) the absence of adapter dimers. Library titers were determined using real time PCR (Kapa Biosystems, Wilmington, MA) on a StepOnePlus Real Time System (ThermoFisher, Waltham, MA). Libraries were mixed to run four samples per lane on the HiSeq 2500 (Illumina). Sequencing was done using a single-read 100-cycle protocol. The resulting base call files (.bcl) were converted to standard fastq formatted sequence files using Bcl2Fastq (Illumina). Sequencing quality was assessed using FastQC (Babraham Bioinformatics, Cambridge, UK). The RNA-seq data is available at NCBI under the accession number GSE102471.

### RNAseq data analysis

To determine the differential expression of genes in LV-GFP and LV-EED containing ARC-ME fragments we used the gene-level edgeR^100^ analysis package. We performed an initial trimming and adapter removal pass using Trimmomatic^101^. Reads that passed Trimmomatic processing were aligned to the rn6 build of the rat genome with Bowtie2/Tophat2^102,103^, and assigned to gene-level genomic features with the Rsubread featureCounts package based on the Ensembl 83 annotation set. Differential expression between LV-GFP and LV-EED injected groups was analyzed using the generalized linear modeling approaches implemented in edgeR^37^. Lists of differentially expressed genes/transcripts were identified based on significance of pairwise comparisons. A subset of genes found to be differentially expressed was selected for subsequent RT-qPCR confirmation.

### WGCNA analysis of RNA-seq data

We used the Weighted-Gene Co-Expression Analysis method for discovery of co-expressed modules of genes in our RNA-seq data. Data were transformed to log2 counts-per-million estimates using the voom function in edgeR. We then determined the 5000 most variable genes across the pooled control and EED-overexpressing samples and applied the WGCNA pipeline to those count estimates. We utilized a signed network and specified a minimum module size of 100 genes; all other parameters were set at defaults. The eigengene summary metric of overall module expression was used to visualize the trend in differential expression of the genes in each module between control and EED- overexpressing samples. We also performed functional enrichment analysis using the DAVID tool^39,40^ for the genes in each WGCNA module. We utilized the human orthologs of these genes as determined by Ensembl for this analysis due to the superior annotation of the human genome; genes that did not have an assigned ortholog were dropped from the analysis. Overrepresented annotation categories for each set of genes were defined as categories with an FDR-value of 5% or less as reported by DAVID’s modified Fisher Exact Test procedure.

### Open array-real time PCR

We used the OpenArray-qPCR platform to measure changes in relative expression for 224 genes studied after perturbing the system by overexpressing EED in the ARC (rats injected on PND21 and euthanized on PND28; ARC-ME fragments collected after a 4 h incubation period to measure changes in pulsatile GnRH release). One thousand ng of total RNA from each ARC-ME fragment were reverse transcribed (RT) using the Omni RT Kit (Qiagen, Valencia, CA) in the presence of random hexamer primers (Invitrogen, Carlsbad, CA), as recommended by the manufacturer. The resulting cDNA was diluted 4 times with H2O and mixed with 2× TaqMan OpenArray Real Time PCR Master Mix (Life Technologies, Grand Island, NY) at a ratio of 3.8:1.2 (PCR mix:cDNA). The mix was loaded into custom made (12 × 224 probes) OpenArray plates (for target genes probe numbers and lengths of amplicon see^20^) using the Quant Studio OpenArray AccuFill platform and the PCR reactions were performed in a QuantStudio 12 K Flex Real-Time PCR System (Applied Biosystems, Foster City, CA).

### Open array analysis

Raw data were extracted from the QuantStudio 12 K Flex software and analyzed using R. CT values were converted to relative expression levels for further analysis using a standard delta-delta transformation. Genes with unusually high variability and/or multiple missing values were dropped at this stage, resulting in 134 genes. We then performed tests for differential expression using the Student’s unpaired T-test; missing values and outliers were dropped from these analyses. Resultant p-values were adjusted for multiple testing using the Benjamini–Hochberg analysis. For further analyses, we imputed missing expression values and the values of removed outliers using k-nearest neighbor imputation using the impute R package before correction.

### Gene network analysis

We utilized a combination of data from the OpenArray platform and standard RT-PCR experiments targeting important genes that were of low quality from the OpenArray runs (e.g. Kiss1) to perform an inference of a network of strong gene co-expression relationships. After correction, strong co-expression networks were derived from the data using a compressive sensing based-method previously described^45^; briefly, the method generates all possible one-gene partial correlation matrices from the data (via the ppcor package in R), then utilizes a CLIME-based approach^104^ as implemented in the R package clime to approximate inverse matrices for each partial correlation matrix. Networks based on a range of values of the regularization parameter lambda for CLIME are generated; we then select a representative network for each inverse partial correlation network based on highest scale-free fit. Co-expression relationships that are preserved in the 95th percentile or more of the distribution of edge preservation in the inverse partial correlation networks after thresholding for interaction strength are included in the overall network. This process was used to construct a network based on the pooled data from controls and from EED-overexpressing samples. Networks were visualized, analyzed and compared using R and Cytoscape 3.1.1 (www.cytoscape.org).

We also utilized the GeneMANIA^53^ gene interaction database to find and visualize known interactions between differentially expressed glutamatergic-related and potassium channel genes located in WGCNA modules of interest, along with the *Kdm6b* second-neighbor genes *Nell2* and *Grm7*. We utilized the human orthologs of these genes for this analysis and the default co-expression, genetic interaction, physical interaction and pathway data sets of the database.

### Chromatin immunoprecipitation (ChIP) assay

To assess the recruitment of EED to putative target gene promoters, and the changes in H3K27me3 association to these promoters caused by EED overexpression, we performed ChIP assays using chromatin extracted from R22 immortalized hypothalamic cells overexpressing EED. The ChIP procedure was carried out essentially as previously described^27,105,106^.

Briefly, cells were washed in ice-cold PBS containing protease a inhibitor cocktail (PI, 1 mM phenylmethylsulfonylfluoride, 7 μg ml^-1^ aprotinin, 0.7 μg ml^−1^ pepstatin A, 0.5 μg ml^−1^ leupeptin), a phosphatase inhibitor cocktail (PhI, 1 mM β-glycerophosphate, 1 mM sodium pyrophosphate and 1 mM sodium fluoride), and an HDAC inhibitor (20 mM sodium butyrate). Cells were cross-linked with 1% formaldehyde for 10 min at room temperature and lysed with 200 µl SDS buffer (0.5% SDS, 50 mM Tris–HCl, 10 mM EDTA) containing protease, phosphatase, and HDAC inhibitors. Chromatin fragmentation was achieved by sonicating the samples for 45 s to yield chromatin fragments of approximately 500 base pairs (bp) using the microtip of a Fisher Scientific FB 705 sonicator. Size fragmentation was confirmed by agarose gel electrophoresis. The sonicated chromatin was clarified by centrifugation at 14,000 rpm for 10 min at 4 °C, brought up to 1 ml in Chip Dilution Buffer (CDB) (16.7 mM Tris–HCl, pH 8.1, 150 mM NaCl, 1.2 mM EDTA, 1.1% Triton X-100, and 0.01% SDS) containing the PI and PhI cocktails, and the HDAC inhibitor described above. The samples were pre-cleared with Protein A/G beads (Dynabeads, Invitrogen, Carlsbad, CA) for 1 h at 4 °C and then stored at − 80 °C. For immunoprecipitation reaction, 50 μl aliquots of chromatin were incubated with 2 μg antibodies listed in Supplementary Table S6. The immunocomplexes were incubated with 25 μl of protein A or G beads solution (Dynabeads) at 4 °C overnight with mild agitation. The next day the beads were washed first with 0.5 ml low salt wash buffer (20 mM Tris–HCl, pH 8.1, 150 mM NaCl, 2 mM EDTA, 1% Triton X-100 and 0.1% SDS), followed by high salt wash buffer (20 mM Tris–HCl, pH 8.1, 500 mM NaCl, 2 mM EDTA, 1% Triton X-100 and 0.1% SDS), LiCl buffer (10 mM Tris–HCl, pH 8.1, 250 M LiCl, 1% Nonidet P-40, 1% sodium deoxycholate and 1 mM EDTA), and finally with TE buffer (10 mM Tris–HCl, pH 8.0 and 1 mM EDTA). Thereafter, the immunocomplexes were eluted with 100 μl of 0.1 M NaHCO3 and 1% SDS at 65 °C for 45 min. Reverse cross-linking was achieved by adding 4 μl of 5 M NaCl and incubating the samples at 95 °C for 30 min. DNA was purified using ChIP DNA Clean & Concentrator columns (Zymo Research, Irvine, CA), and stored at − 80 °C until qPCR analysis. All the chemicals were purchased from Sigma-Aldrich (St. Louis, MO, USA).

### qPCR detection of chromatin immunoprecipitated DNA

The proximal promoter regions of the genes of interest were amplified by qPCR. Supplementary Table S5 lists the accession numbers of the genes analyzed, as well as the chromosomal position of the 5′-flanking regions amplified, using the position of the transcription start site (TSS) as the reference point. The primer sequences (Eurofins MWG Operon, Huntsville, AL) used to amplify immunoprecipitated DNA fragments are also shown in Supplementary Table S5. PCR reactions were performed using 1 µl of each immunoprecipitate (IP) or input samples (see below), primer mix (1 µM each primer), and SYBR Green Power Up Master Mix (Thermo Fisher, Waltham, MA) in a final volume of 10 µl. Input samples consisted of 10% of the chromatin volume used for immunoprecipitation. The thermocycling conditions used were: 95 °C for 5 min, followed by 40 cycles of 15 s at 95 °C and 60 s at 60 °C. Data are expressed as % of IP signal/Input signal.

### Statistics

Statistical analyses and figures were created with Prism 9 (GraphPad Software, San Diego, CA, USA, www.graphpad.com”). The differences between several groups were analyzed by one-way ANOVA followed by the Student–Newman–Keuls multiple comparison test for unequal replications. The Student’s t test was used to compare two groups. When comparing percentages, groups were subjected to arc–sine transformation before statistical analysis to convert them from a binomial to a normal distribution^107^. A p value of < 0.05 was considered statistically significant.

## Supplementary Information

Supplementary Legends.

Supplementary Figure S1.

Supplementary Figure S2.

Supplementary Figure S3.

Supplementary Figure S4.

Supplementary Figure S5.

Supplementary Table S1.

Supplementary Table S2.

Supplementary Table S3.

Supplementary Table S4.

Supplementary Table S5.

Supplementary Table S6.
