# Supplementary material for: Polycomb represses a gene network controlling puberty via modulation of histone demethylase Kdm6b expression
Source: Sci Rep. 2021 Jan 21;11:1996. doi: 10.1038/s41598-021-81689-4 (PMC7819995; doi:10.1038/s41598-021-81689-4)
Supplement: Supplementary file 1 — Supplementary Legends. [file 41598_2021_81689_MOESM1_ESM.docx]

**Supplementary Figure 1: GeneMANIA networks of glutamatergic and potassium channel genes downregulated by EED.** Network of previously-reported relationships between the glutamatergic-related genes *Nell2* and *Grm7* (yellow nodes) with glutamatergic and potassium channel genes downregulated by EED-overexpression (cyan nodes), detected by either RNA-seq or OpenArray, and shown as reported by the GeneMANIA database query tool. Blue lines indicate co-expression, green lines indicate genetic interactions and orange lines indicate known pathway interactions.

**Supplementary Figure 2: Changes in gene expression elicited by EED overexpression in the ARC.** Loss of expression of glutamatergic-related and potassium channel genes classified in modules 2, 3, 6 and 7 by WGCNA analysis, after overexpressing EED in the ARC of immature female rats. The animals received a bilateral injection of LV-GFP or LV-EED in the ARC at the beginning of juvenile development (22-days of age), and the MBH was collected on postnatal day 28; mRNA levels were measured by qPCR. Results are expressed as fold change with respect to control values. * = p < 0.05, *** p < 0.001 vs. LV-GFP- Control treated rats. (Student’s t-test) (n=8 per group).

**Supplementary Figure 3: Changes in gene expression elicited by EED overexpression in hypothalamic R22 cells.** Loss of expression of glutamatergic-related and potassium channel genes in hypothalamic R22 cells. Cells were infected with of LV-GFP or LV-EED, isolated by FACS and expanded prior mRNA levels measured by qPCR. Results are expressed as fold change with respect to control values. * = p < 0.05, ** p < 0.01, *** p < 0.001 vs. LV-GFP- Control infected cells (Student’s t-test) (n=4 per group). N.D.= not detectable.

**Supplementary Figure 4: Changes in EED and H3K27me3 recruitment to the promoter of network genes.** (**A**) Recruitment of EED to the promoters of glutamatergic-related and potassium channel genes in R22 cells overexpressing EED. (**B**) Increased H3K27me3 abundance at the promoter of glutamatergic-related and potassium channel genes in R22 cells after EED overexpression. Results are expressed as fold-change with respect to cells transduced with LV-GFP. * = p < 0.05, ** = p < 0.01 vs. LV-GFP treated infected (Student’s t-test) (n=4 per group).

**Supplementary Figure 5: KDM6B partially counteracts the effects of EED on selected glutamatergic-related and potassium channel genes.** Rat R22 cells were transiently transfected with rat *Eed*, a human *KDM6B* expression vector or both. Gene expression was determined forty-eight hours after transfection. mRNA levels were measured by qPCR. Results are expressed as fold change with respect to control-pcDNA values. Vs pcDNA- Control treated cells (Student-Newman-Keuls test) (n=3 per group).

**Supplementary Table 1:** DAVID functional analysis of genes in modules from WGCNA of the rat ARC nucleus.

**Supplementary Table 2:** Differential expression analysis of RNAseq data from LV-Control and LV-EED rat ARC nucleus.

**Supplementary Table 3:** Differential expression analysis of custom made OpenArray data from LV-Control and LV-EED rat ARC nucleus.

**Supplementary Table 4:** Partial correlation analysis of ARC nucleus gene expression with GnRH release.

**Supplementary Table 5:** Primers used for OpenArray, RT-qPCR and CHIP-PCR.

**Supplementary Table 6:** Antibodies used for CHIP assays.
