# Supplementary figures and images for: Polycomb represses a gene network controlling puberty via modulation of histone demethylase Kdm6b expression
Source: Sci Rep. 2021 Jan 21;11:1996. doi: 10.1038/s41598-021-81689-4 (PMC7819995; doi:10.1038/s41598-021-81689-4)

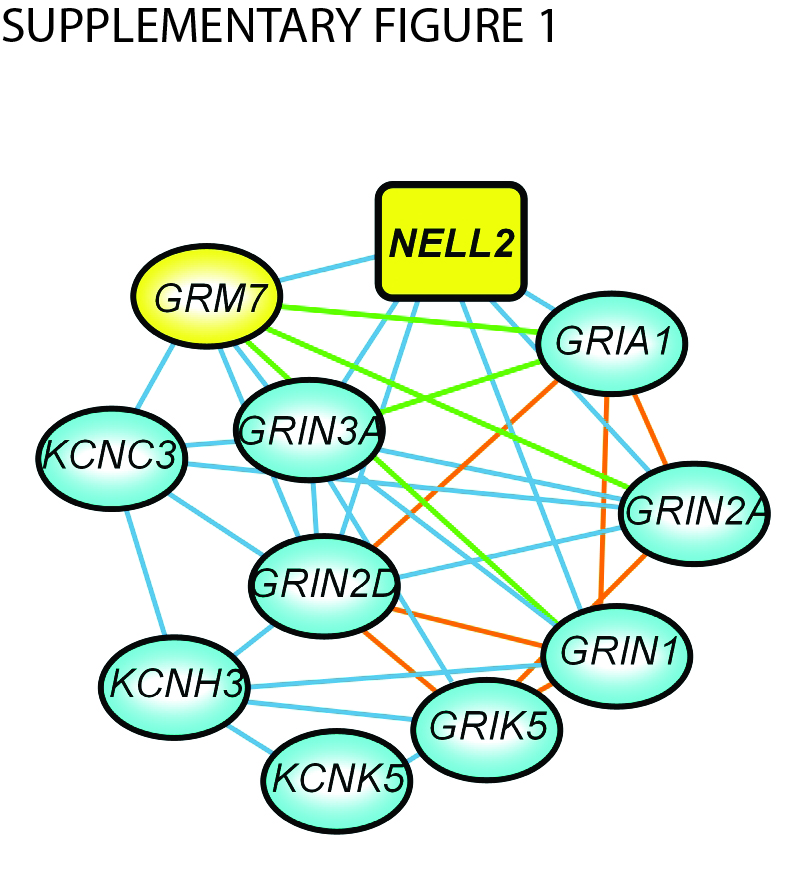

Supplement: Supplementary file 2 — Supplementary Figure S1. [file 41598_2021_81689_MOESM2_ESM.jpg]

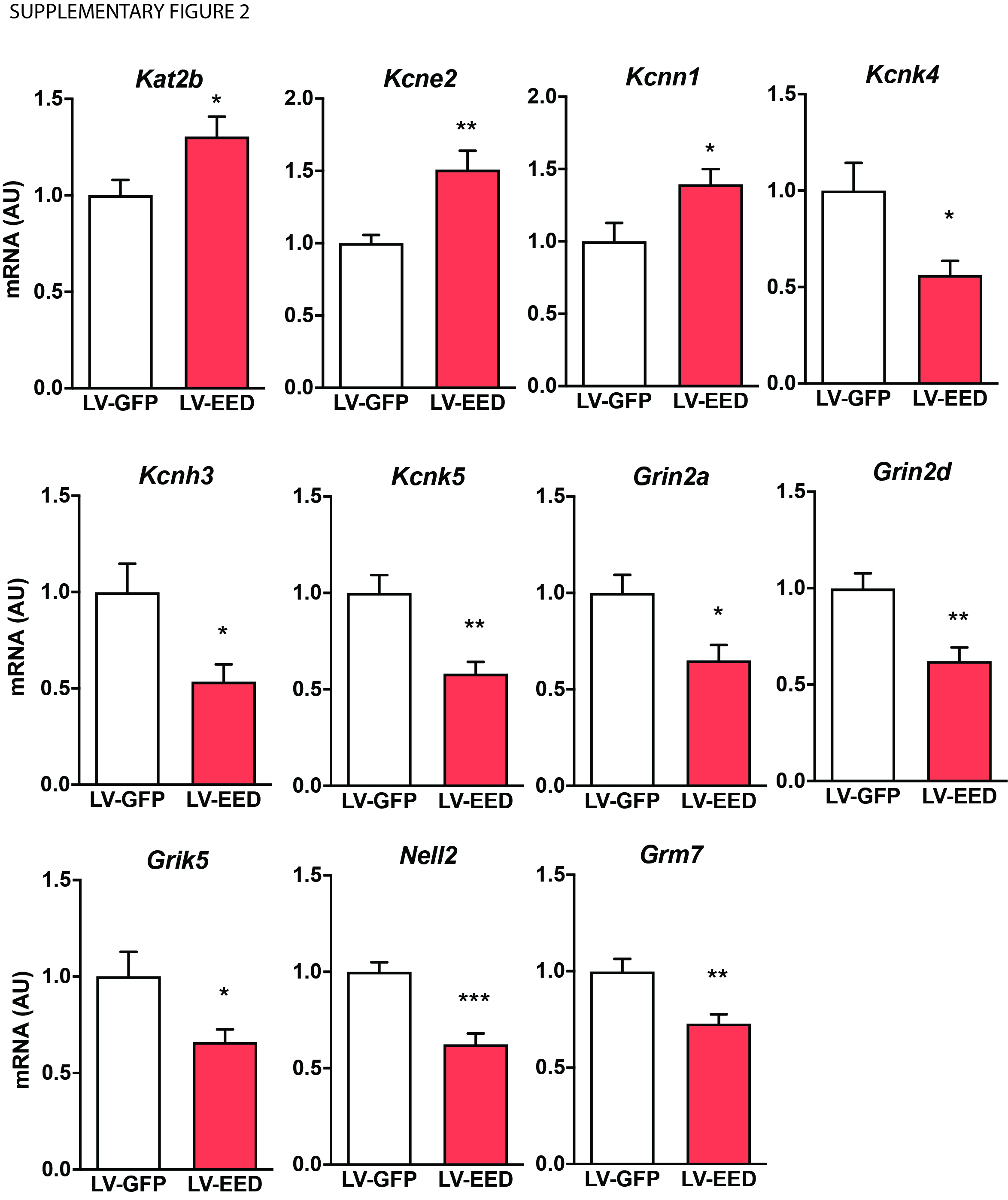

Supplement: Supplementary file 3 — Supplementary Figure S2. [file 41598_2021_81689_MOESM3_ESM.jpg]

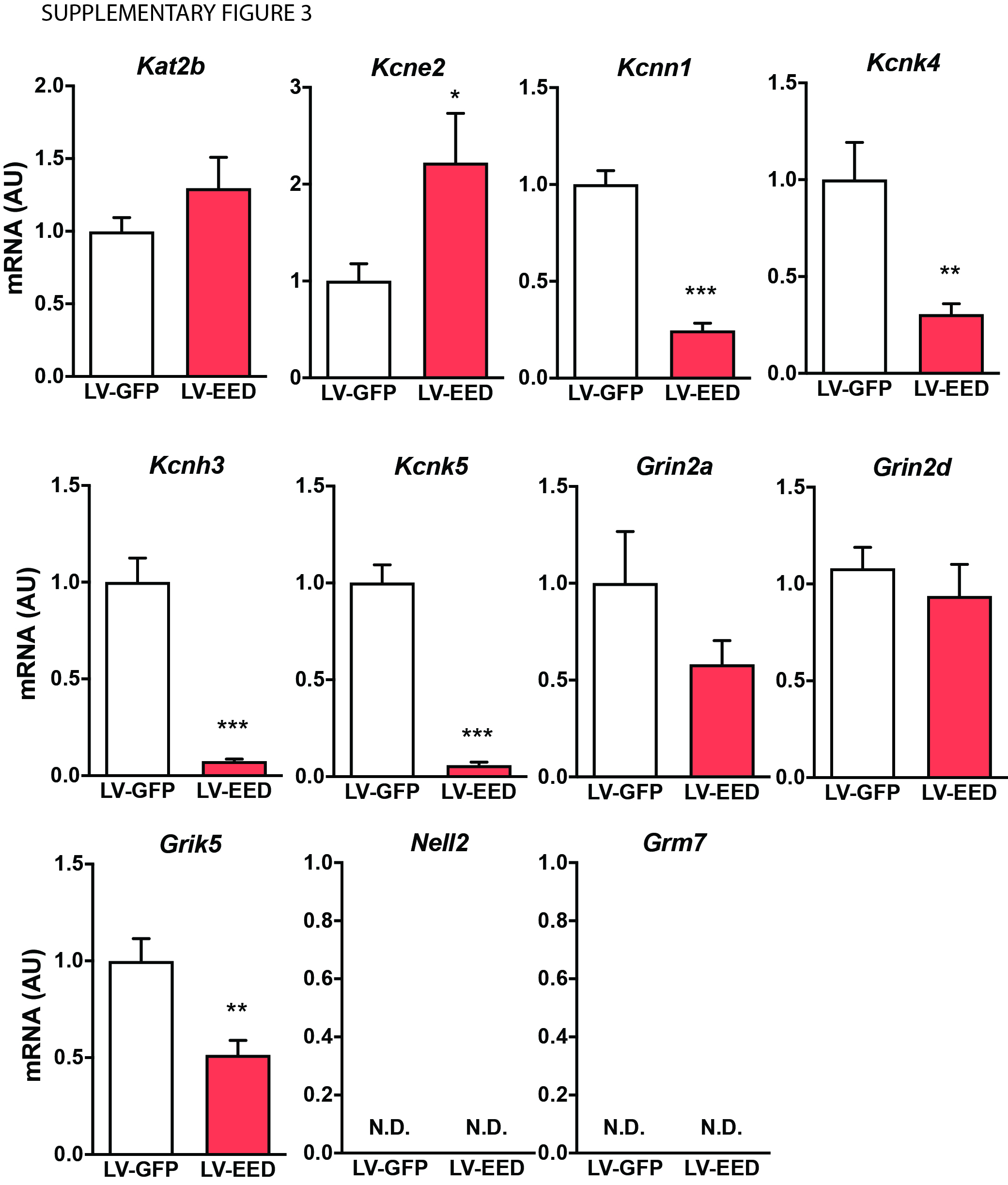

Supplement: Supplementary file 4 — Supplementary Figure S3. [file 41598_2021_81689_MOESM4_ESM.jpg]

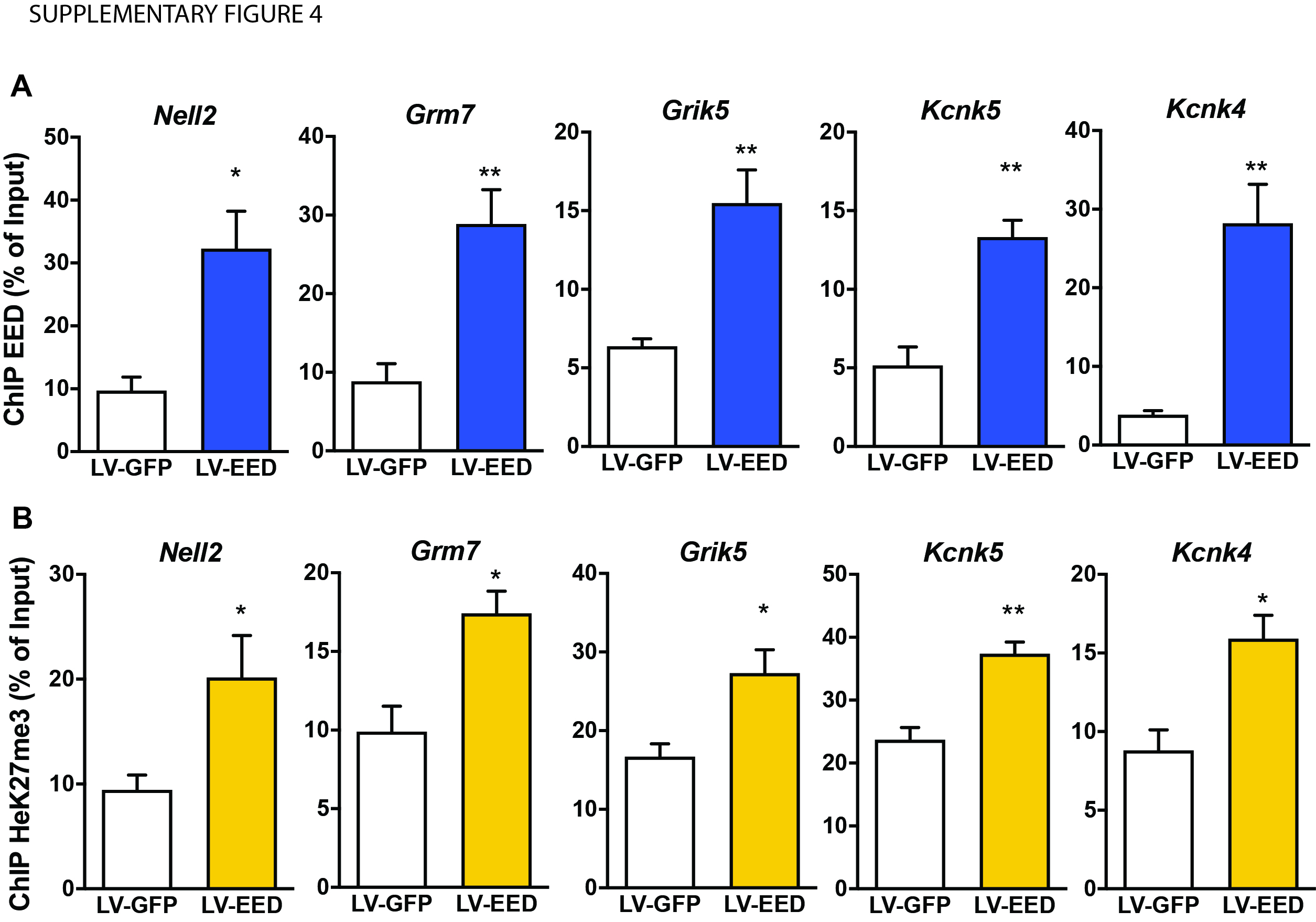

Supplement: Supplementary file 5 — Supplementary Figure S4. [file 41598_2021_81689_MOESM5_ESM.jpg]

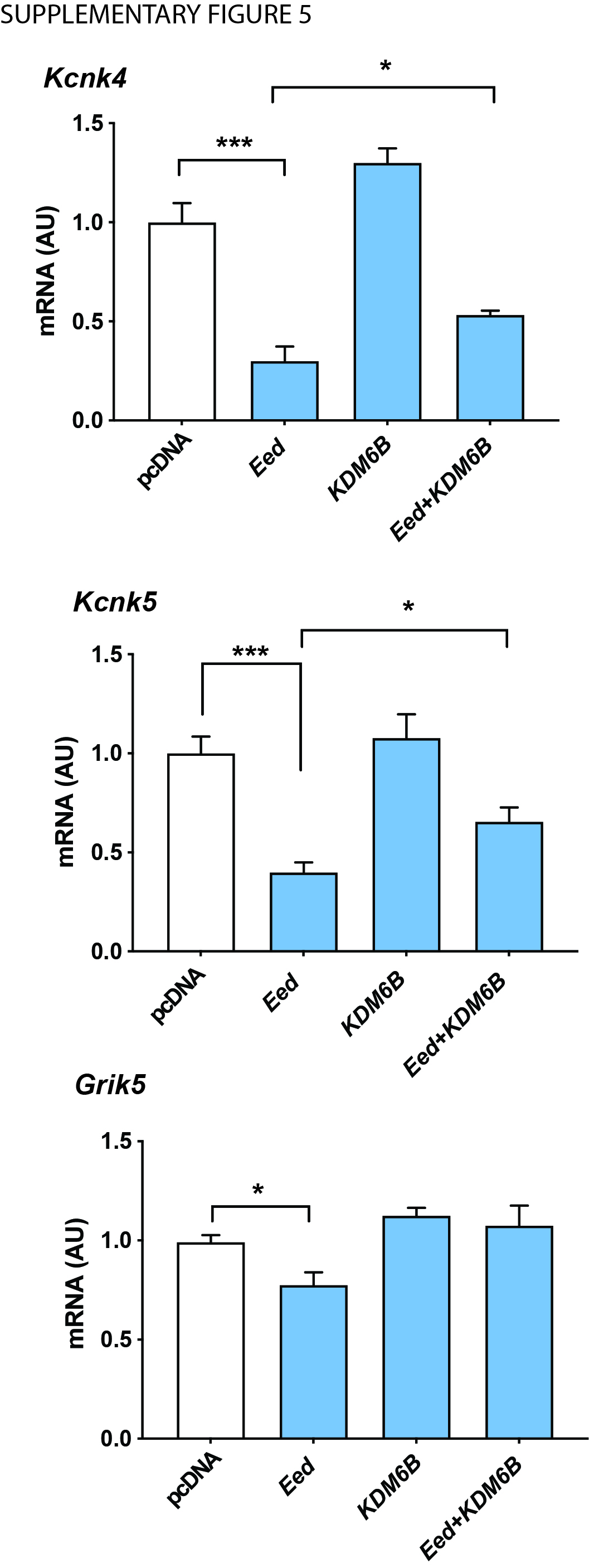

Supplement: Supplementary file 6 — Supplementary Figure S5. [file 41598_2021_81689_MOESM6_ESM.jpg]
